# The Effect of Varying Almond Shell Flour (ASF) Loading in Composites with Poly(Butylene Succinate (PBS) Matrix Compatibilized with Maleinized Linseed Oil (MLO)

**DOI:** 10.3390/ma11112179

**Published:** 2018-11-03

**Authors:** Patricia Liminana, Luis Quiles-Carrillo, Teodomiro Boronat, Rafael Balart, Nestor Montanes

**Affiliations:** Technological Institute of Materials (ITM), Universitat Politècnica de València (UPV), Plaza Ferrándiz y Carbonell 1, 03801 Alcoy, Spain; patligre@mcm.upv.es (P.L.); tboronat@dimm.upv.es (T.B.); rbalart@mcm.upv.es (R.B.); nesmonmu@upvnet.upv.es (N.M.)

**Keywords:** green composites, natural fillers, poly(butylene succinate) (PBS), almond shell flour (ASF)

## Abstract

In this work poly(butylene succinate) (PBS) composites with varying loads of almond shell flour (ASF) in the 10–50 wt % were manufactured by extrusion and subsequent injection molding thus showing the feasibility of these combined manufacturing processes for composites up to 50 wt % ASF. A vegetable oil-derived compatibilizer, maleinized linseed oil (MLO), was used in PBS/ASF composites with a constant ASF to MLO (wt/wt) ratio of 10.0:1.5. Mechanical properties of PBS/ASF/MLO composites were obtained by standard tensile, hardness, and impact tests. The morphology of these composites was studied by field emission scanning electron microscopy—FESEM) and the main thermal properties were obtained by differential scanning calorimetry (DSC), dynamical mechanical-thermal analysis (DMTA), thermomechanical analysis (TMA), and thermogravimetry (TGA). As the ASF loading increased, a decrease in maximum tensile strength could be detected due to the presence of ASF filler and a plasticization effect provided by MLO which also provided a compatibilization effect due to the interaction of succinic anhydride polar groups contained in MLO with hydroxyl groups in both PBS (hydroxyl terminal groups) and ASF (hydroxyl groups in cellulose). FESEM study reveals a positive contribution of MLO to embed ASF particles into the PBS matrix, thus leading to balanced mechanical properties. Varying ASF loading on PBS composites represents an environmentally-friendly solution to broaden PBS uses at the industrial level while the use of MLO contributes to overcome or minimize the lack of interaction between the hydrophobic PBS matrix and the highly hydrophilic ASF filler.

## 1. Introduction

Over the last years, research on new polymer materials has attracted much research with the aim of minimizing the environmental impact of petroleum-derived polymers. These new polymers, also known as biopolymers, have demonstrated a clear contribution to decrease the carbon footprint in comparison to conventional plastics [1,2]. High environmentally-friendly polymers can be obtained from renewable resources and can potentially find interesting engineering applications. These biobased polymers include polysaccharides (cellulose, starch, chitosan, and so on), protein polymers (gluten, ovalbumin, soy protein, collagen, among others), and bacterial polymers such as poly(3-hydroxybutyrate), PHB, and other polymers obtained from biomass fermentation by different microorganisms [3,4,5,6,7]. Some polymers can be obtained from petroleum resources, but they show high environmental efficiency at the end-of-life as they can undergo full disintegration under certain conditions (compost). Aliphatic polyesters such as poly(butylene succinate) (PBS), poly(glycolic acid) (PGA), poly(ε-caprolactone) (PCL), poly(butylene succinate*-co-*adipate) (PBSA), and some aliphatic-aromatic copolyesters, i.e., poly(butylene succinate*-co-*terephthalate) (PBAT), poly(butylene succinate*-co-*terephthalate) (PBST), among others, belong to these petroleum-based, disintegrable (biodegradable) polymers [8,9]. 

Among these polyesters PBS is gaining relevance due to its high flexibility which allows its use in the packaging industry. Poly(butylene succinate) can be obtained from polycondensation of succinic acid and 1,4-butanediol (BDO). Although the most common route to obtain PBS is from petroleum-derived monomers (in fact, the first PBS commercial grades were petroleum-derived), currently it is possible to obtain both starting monomers from renewable resources such as starch, glucose or cellulose by bacterial fermentation [10,11], and this will open a new age in the development of biopolyesters from renewable resources. Obviously, bio-derived PBS is a high environmentally-friendly material, from both points of view: origin (bio-derived) and end-of-life (disintegrable in controlled compost soil). Nevertheless, petroleum-derived PBS lacks the “bio” origin, but it does not generate problematics at the end-of-life since, as other aliphatic polyesters, it can undergo disintegration in controlled compost soil [12]. So, currently, petroleum-based PBS and derivatives are interesting alternatives to other non-biodegradable plastics. Recently, Puchalski et al. have reported the degradation of petroleum-derived PBS and PBSA subjected to different environmental conditions. In addition they reported the change in physical and mechanical properties of PBS and PBSA during the degradation processes [13].

Poly(butylene succinate) possesses comparable properties to those of some commodities such as poly(ethylene) (PE) or poly(propylene) (PP) [14]. In addition, processing of PBS can be carried out at moderate temperatures, with a pre-drying stage to remove moisture, which is responsible for hydrolysis. Its main uses include film/sheet for the packaging and agricultural industries [15,16]; despite this, its use is increasing in the automotive industry and medical devices as well [17,18,19]. The main drawback of PBS is its high cost compared to commodity and some engineering plastics.

One way to partially overcome this drawback without compromising its biodegradability is by blending it with less expensive biodegradable polymers such as poly(lactic acid) (PLA), PCL, among others [20,21]. Another approach is by using lignocellulosic fillers to give the so-called natural polymer composites (NPCs) with PBS matrix. Natural polymer composites can positively contribute to give sustainable materials with balanced properties (mechanical, thermal, barrier, physical, and so on), similar to commodities [22,23,24]. An interesting approach to these fillers is the use of industrial or agroforestry by-products to act as reinforcing fillers in NPCs. It has been widely reported the potential of industrial wastes from the food industry (fruit shells, stalks, fruit skins, seeds, among others) in NPCs [18,25,26,27]. 

Poly(butylene succinate) has been successfully used as matrix with a wide variety of natural fibers such as sisal [28], hemp [29], kenaf [30], and so on. Regarding PBS composites with agricultural wastes, it is worthy to note the work by Tserki et al. [31], in which, lignocellulosic waste flours (spruce, olive husk and paper) were used as fillers into PBS matrices. Yeng et al. [32] reported the use of fillers from wheat bran into modified PBS matrices. They reported the positive effect of grafting maleic anhydride into PBS chains to give poly(butylene succinate*-g-*maleic anhydride) which showed increased interactions with the cellulosic components contained in the wheat bran.

Among the wide variety of potential lignocellulosic wastes, almond shell is an abundant waste in countries such as Spain, which stands as the third major producer of almonds just after the USA and Australia. Almond shell has been employed as lignocellulosic fillers in several NPCs. In particular, it has been added to commodities such as PP [33,34], but moreover, several studies involving almond shell wastes and some biopolymers, PLA [35,36] or PCL [37], have been reported in the last years. In a previous work [38], our group reported the need of compatibilizer agents to provide increased interactions between the highly hydrophobic PBS matrix and the highly hydrophilic almond shell flour (ASF) filler as observed in other polymer/lignocellulosic filler composites [39,40]. Several solutions to overcome the low polymer/filler interactions have been proposed such as silane treatments, acetylation, plasma treatments, and so on [41,42,43]. Excellent results have been obtained by using maleic anhydride grafted copolymers in both polymer/lignocellulosic fillers and binary/ternary blends with immiscible polymers [44,45]. Recently, vegetable oils have been proposed as environmentally-friendly compatibilizers as an alternative to conventional petroleum-based ones [46,47]. Epoxidized vegetable oils (EVOs), such as epoxidized soybean oil (ESBO), epoxidized linseed oil (ELO) [48,49], epoxidized palm oil (EPO) [50], and so on, have been successfully used as compatibilizers in NPCs. Another vegetable oil derivative, namely maleinized linseed oil (MLO) has been used as compatibilizers in polymer/lignocellulosic composites [51,52]. Ferri et al. [53] reported a clear improvement on processability, mechanical ductile properties and thermal stability on PLA composites with low MLO loading content. In our previous work, focused on PBS/ASF composites with a constant ASF content of 30 wt %, several compatibilizer families based on different reactive groups, i.e., epoxy, maleic anhydride and acrylic were used. Maleinized linseed oil—MLO—gave the best results in terms of balanced properties due to the reaction of the succinic anhydride group attached to the triglyceride molecule, towards the hydroxyl groups contained in both PBS (end-chain groups) and ASF (cellulose and hemicellulose) [38]. 

The aim of this work is to expand the potential of PBS/ASF composites by varying the ASF wt % loading up to 50 wt % and using MLO as reactive compatibilizer.

## 2. Materials and Methods 

### 2.1. Materials

Poly(butylene succinate)-based composites were manufactured with a PBS commercial grade Bionolle 1020MD supplied by Showa Denko Europe GmbH (Munich, Germany). This PBS grade is petroleum-derived but fully disintegrable in controlled compost soil. This PBS possesses a melt flow index—MFI comprised between 20 and 34 g/10 min and a density of 1.26 g cm^−3^. The lignocellulosic filler was almond shell supplied by JESOL Materias Primas (Valencia, Spain). The almond shell was grinded and sieved in a CISA®RP09 sieve shaker (CISA Cedacería Industrial, Barecelona, Spain) to an average particle size of 150 μm. The selected compatibilizer was maleinized linseed oil—MLO, VEOMER LIN supplied by Vandeputte (Mouscron, Belgium). This modified vegetable oil is characterized by a viscosity of 10 dPa s at 20 °C and an acid value comprised in the 105–130 mg KOH g^−1^ range.

### 2.2. Manufacturing of PBS/ASF/MLO Composites

As polyesters are very sensitive to hydrolysis, PBS was previously dried at 50 °C for 24 h to avoid degradation during processing. The ASF was also dried in the same conditions as PBS in a dehumidifying dryer MDEO from Industrial Marsé (Barcelona, Spain). The MLO was heated at 40 °C for 30 min to reduce its viscosity and enhance mixing with both PBS and ASF.

Table 1 shows the formulations developed in this study. The appropriate amounts of each component were weighed and mechanically pre-mixed in a zipper bag for 5 min. Then, the mixtures were compounded in a twin-screw co-rotating extruder from Construcciones Mecánicas DUPRA, S.L. (Alicante, Spain). The rotation speed was set to 40 rpm and the temperature of the four heated barrels was programmed to 120 °C (hopper), 125 °C, 130 °C, and 130 °C (dye). The screws had a diameter of 25 mm and a length-L to diameter-D ratio of 24. After extrusion, the obtained compounds were pelletized and dried again at 50 °C for 24 h before further processing in a Meteor 270/75 injection moulding machine from Mateu&Sole (Barcelona, Spain). The temperature profile was set to (from the hopper to the injection nozzle): 110 °C, 115 °C, 130 °C, and 125 °C.

### 2.3. Mechanical Characterization

PBS/ASF/MLO composites were characterized by standard tensile tests following ISO 527-1:2012 in a universal test machine ELIB 50 from S.A.E. Ibertest (Madrid, Spain). The load cell was 5 kN and the crosshead speed was set to 10 mm min^−1^. In addition, Shore D hardness values of PBS/ASF/MLO composites were obtained in a durometer model 676-D from J. Bot Instruments (Barcelona, Spain) as indicated in ISO 868:2003. Finally, the impact strength was obtained using the Charpy pendulum (with an energy of 1 J) on notched samples (“V” type notch with a radius of 0.25 mm), according to ISO 179-1:2010. At least five different samples were used in each mechanical test and the average values of the corresponding parameters were obtained. In particular, the elongation at break—*ε*_b_, maximum tensile strength—*σ*_t_ and the tensile modulus—E_t_, were obtained from tensile tests. All the tests were conducted at room temperature.

### 2.4. Thermal and Thermomechanical Characterization

Differential scanning calorimetry (DSC) was used to study the main thermal transitions of PBS/ASF/MLO composites in a model DSC 821 calorimeter from Mettler-Toledo (Schwerzenbach, Switzerland). The average sample weight was comprised between 5 and 7 mg and standard aluminium crucibles with a volume of 40 μL were used. All the samples were subjected to a thermal program consisting on three stages. The first stage consisted on a first heating cycle from 25 °C up to 200 °C. Then, a cooling stage down to −50 °C was applied, and finally, a second heating stage from −50 °C up to 300 °C was scheduled. The heating/cooling rate was 10 °C min^−1^ for all three stages and a constant nitrogen flow of 66 mL min^−1^ was used. All DSC tests were run in triplicate to obtain reliable results. The degree of crystallinity (*χ_c_*) was calculated using the following equation:

(1) $$\%\chi_{c}=\left[\frac{\Delta H_{m}}{\Delta H_{m}^{0}\cdot \left(1-w\right)}\right]\cdot 100\;$$

where $\Delta H_{m}$ corresponds to the measured melt enthalpy of PBS. $\Delta H_{m}^{0}$ (J g^−1^) stands for the theoretical melt enthalpy of a fully crystalline PBS, which was taken as 110.3 J g^−1^ for PBS as previously reported [54]. Regarding *w*, it represents the total weight percent of all components (ASF and MLO) added to PBS matrix.

Thermal stability of PBS/ASF/MLO composites was studied by thermogravimetry in a TGA/SDTA 851 thermobalance from Mettler-Toledo (Schwerzenbach, Switzerland). The average sample weight for TGA characterization was in the 7–10 mg range and standard alumina crucibles with a total volume of 70 μL were used. The scheduled thermal program was a dynamic heating from 30 °C up to 700 °C at a heating rate of 20 °C min^−1^ in air atmosphere.

Thermomechanical characterization was carried out by dynamic mechanical thermal analysis (DMTA) in a Mettler-Tolledo DMA1 (Schwerzenbach, Switzerland). Samples were subjected to a dynamic flexural test in single cantilever at a frequency of 1 Hz. The thermal program consisted on a heating sweep from −50 °C up to 80 °C at a constant heating rate of 2 °C min^−1^. The maximum flexural deformation was set to 10 μm. On the other hand, the thermal/dimensional stability was determined in a thermomechanical analyzer—TMA Q400 from TA Instruments (Delaware, USA). Squared samples with parallel faces sizing 4 × 10 × 10 mm^3^ were subjected to a constant load of 0.02 N and subsequently subjected to a heating program from −50 °C to 80 °C at a heating rate of 2 °C min^−1^. The coefficient of linear thermal expansion—CLTE was calculated below and over the glass transition temperature, *T_g_*.

### 2.5. Morphology Characterization

The morphology of PBS/ASF/MLO composites was studied by field emission scanning electron microscopy (FESEM) in a FESEM model ZEISS ULTRA 55 from Oxford Instruments (Abingdon, UK). Fractured samples from impact tests were subjected to a sputtering process with an aurum-palladium alloy inside a sputter coater model EMITECH SC7620 from Quorum Technologies (East Sussex, UK).

## 3. Results and Discussion

### 3.1. Appearance and Mechanical Properties of PBS/ASF/MLO Composites.

**Figure 1:**
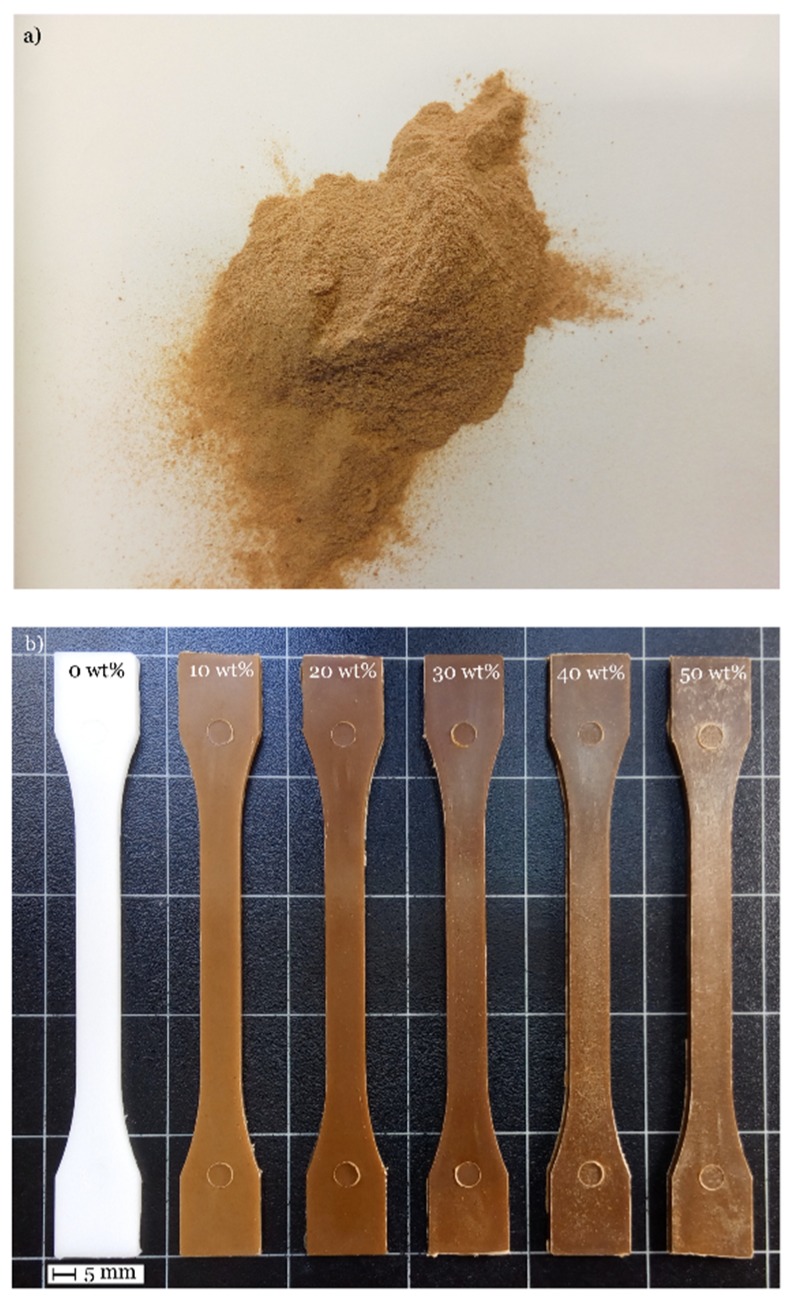
(**a**) Powdered almond shell flour (ASF) and (**b**) injection moulded PBS/ASF/MLO composites with varying ASF content in wt %.

As observed in Figure 1, neat PBS is white due to its semicrystalline nature [13,55]. As the ASF loading increases, we can observe a slight change in color but in general, their appearance is like other wood plastic composites.

**Figure 2:**
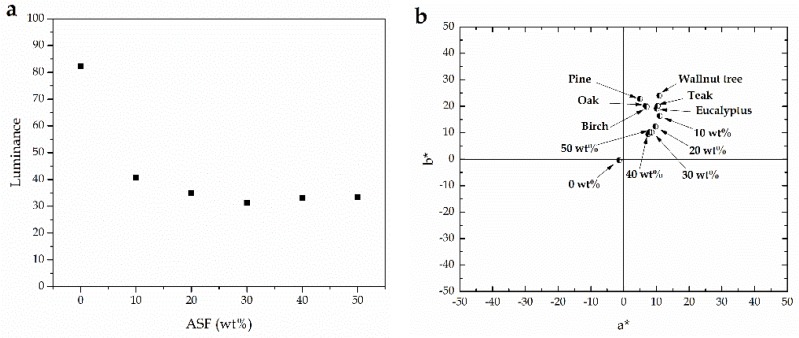
(**a**) Variation of the luminance (L*) and (**b**) colour coordinates (a*b*) of PBS/ASF/MLO composites with varying ASF loading.

As the ASF increases, the material becomes darker and this can be followed by the evolution of the luminance (L*) of the samples as observed in Figure 2a. In addition, the colour coordinates a* and b* offer the real changes in brownish colour. The a* coordinate changes from negative values (green) to positive values (red) while the b* coordinate provides a measurement of the change in colour from blue (negative values) to yellow (positive values). As it can be seen in Figure 2b, all samples are placed in the a* > 0 and b* > 0 quadrant. In particular, both the a* and b* coordinates decrease as the ASF loading increases. Figure 2b also shows the colour coordinates (a*b*) of several commercial woods [56,57]. Poly(butylene succinate)/ASF/MLO composites with 10 wt % show similar colour coordinates to those of eucalypt [58] and teak woods [59]. As the ASF content increases, the yellow content decreases and the obtained materials are browner.

Typically, the addition of an uncompatibilized lignocellulosic filler into a polymer matrix, leads to a decrease in mechanical properties due to the low polymer/particle interactions. This lack (or very poor) interactions results in a decrease in material’s cohesion which gives decreased elongation at break—*ε_b_* and maximum tensile strength—*σ_t_* as they are very sensitive to cohesion. In contrast, the material usually becomes stiffer with increased modulus as this represents the stress to strain ratio in the linear region [60]. Individual MLO additive into a thermoplastic polyester matrix, acts with several overlapping effects. The first one is a typical plasticization effect which gives a slight decrease in *T_g_* with the subsequent decrease in all mechanical resistant properties (maximum tensile strength and modulus), while the elongation at break is increased. A second effect of MLO is chain extension due to reactions of the attached succinic anhydride group with hydroxyl groups located on polyester end-chains which gives increased elongation at break [52,53]. In this work, addition of both ASF and MLO should lead to overlapping all the above-mentioned phenomena in a complex way. Figure S1 in Supplementary Information shows a plot comparison of the typical stress (*σ*)—strain (*ε*) curves for PBS/ASF/MLO composites with different ASF and MLO loading. Table 2 summarizes the main mechanical properties of PBS/ASF/MLO composites. As it can be seen, a progressive decrease in maximum tensile strength from neat PBS (31.5 MPa) down to very low values of 7.1 MPa (50 wt % ASF + 7.5 MLO) are obtained. This dramatic decrease in tensile strength is directly related to the large filler content (in fact, the PBS polymer matrix only represents 42.5 wt % in composites with 50 wt % ASF). With regard to elongation at break—*ε_b_*, the behavior is quite interesting. PBS is a very flexible polymer with a *ε_b_* of 215.6%. The only addition of uncompatibilized 30 wt % ASF leads to a dramatic decrease in *ε_b_* down to 6.3% as reported in our previous work [38], and this value was remarkably improved up to 25.8% by compatibilization with 4.5 wt % MLO. A decrease in *ε_b_* is typical of uncompatibilized particle-filled polymers. The lack (or poor) polymer-particle interactions are responsible for this high decrease. It is important to remark that lignocellulosic fillers are high hydrophilic due to their cellulose and hemicellulose content while, on the other hand, most polymers are hydrophobic. These extremely high difference in polarity is responsible for poor polymer-particle interactions and this leads to poor material cohesion. As *ε_b_* is highly sensitive to cohesion, uncompatibilized composites with a polymer matrix and a lignocellulosic filler, show a dramatic decrease in *ε_b_* values. Despite some petroleum-derived compatibilizers (mainly copolymers) have been widely used to increase polymer-particle filler interactions, ε_b_ is not remarkably improved but in this work, the use of a flexible molecule derived from a natural triglyceride, allows overcoming or minimizing this poor polymer-particle interaction thus allowing a moderate increase in *ε_b_*, with regard to uncompatibilized PBS/ASF composites [61,62,63,64]. It seems evident that MLO addition provides improved ductile properties to PBS/ASF composite with 30 wt % ASF. In fact, the *ε_b_* is higher than 16% for all composites, depending on the ASF and MLO content. For low ASF loading (up to 30 wt %) an increasing tendency in *ε_b_* can be observed. As indicated previously, the particle filler provides a remarkable decrease in *ε_b_* while individual MLO gives increased elongation due to different phenomena. The positive compatibilizing effect of MLO is evident for these concentrations, with increasing *ε_b_* as MLO content increases from 1.5 up to 4.5 wt %. In fact, the compatibilized composite with 30 wt % ASF and 4.5 wt % MLO gives the highest *ε_b_* value. It seems that there is an ASF threshold at 30 wt %. Below this threshold, MLO can effectively compatibilize PBS/ASF/MLO composites in a complex process which involves several MLO mechanisms. Over 30 wt % ASF, a decrease in *ε_b_* down to 16% is obtained. This could mean that once the compatibilization threshold has been overpassed, compatibilization does not occur in a correct way (maybe due to the high filler content). As indicated previously, MLO could be responsible for some overlapping phenomena and, moreover, as it is not fully miscible with polyesters, plasticizer saturation could occur as reported previously with polymers and composites with modified vegetable oils [65]. The evolution of the tensile modulus also suggests that there is an ASF threshold at about 30 wt % above which, compatibilization does not occur in an appropriate way.

With regard to Shore D hardness values, addition of ASF offers the same tendency than that observed for elongation at break but the changes are not significant. Regarding the impact strength, PBS/ASF/MLO composites show interesting behavior. As reported in our previous work, the addition of 30 wt % ASF to PBS without any compatibilizer gives an impact energy of 1.8 kJ m^−2^, which is increased up to double by the addition of 4.5 wt % MLO (3.8 kJ m^−2^) [38]. It is important to remark that the impact strength is directly related to mechanical resistant properties and ductile properties as well. So that, both an increase in tensile strength and an increase in elongation at break are representative for improved impact strength. So, by taking into account this fact, a decreasing tendency can be observed for impact strength with values of 5.4 kJ m^−2^ for PBS/ASF/MLO composites with 10 wt % ASF down to values of 2.6 kJ m^−2^ for the composite with the highest ASF content (50 wt %). It is worthy to note that even for this high ASF content, the impact strength is remarkably higher than that of the uncompatibilized composite consisting on PBS and 30 wt % ASF.

**Figure 3:**
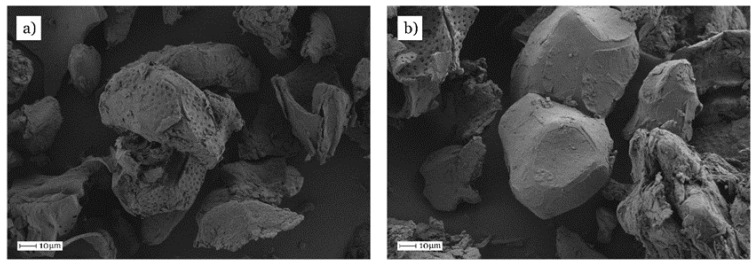
Field emission scanning electron microscopy (FESEM) images (500×) corresponding to almond shell flour (ASF) particles, (**a**) with spotted surfaces and porous structure and (**b**) with a smooth surface.

This particular mechanical response can be understood by evaluating the fracture surfaces from impact tests. Figure 3 shows the typical shape of almond shell flour (ASF) microparticles. As one can see, it is possible to find the typical spotted surface of almond shell on isolated micro-particles (Figure 3a). These particles provide a porous structure that can be positive for polymer/particle interactions. In addition to these shapes, it is possible to find rounded particles with smoothed surface a lower porosity (Figure 3b).

**Figure 4:**
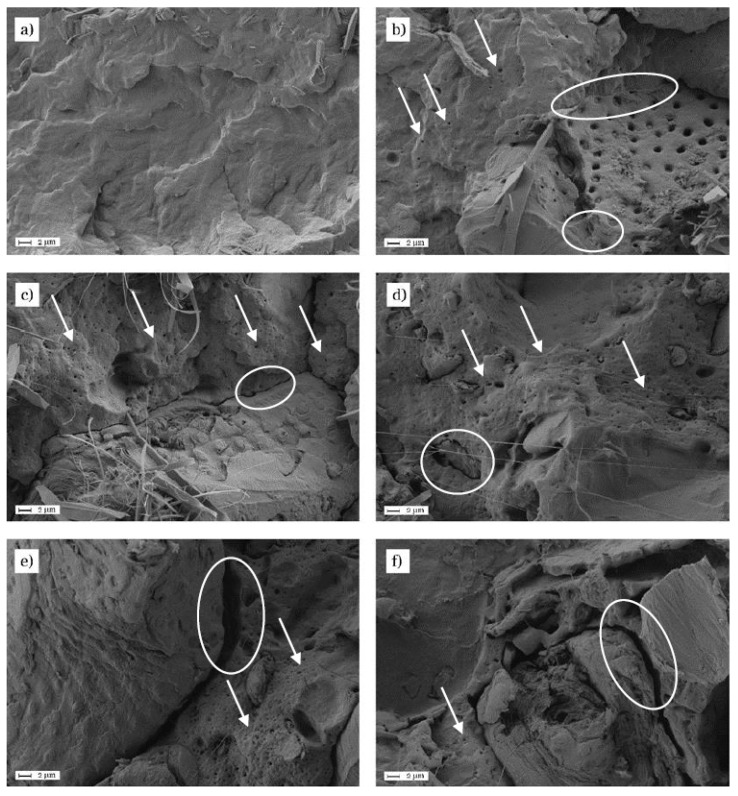
Field emission scanning electron microscopy (FESEM) images (2000×) of PBS/ASF/MLO composites with varying ASF content: (**a**) neat PBS; (**b**) 10 wt % ASF, 1.5 wt % MLO; (**c**) 20 wt % ASF, 3.0 wt %; (**d**) 30 wt % ASF, 4.5 wt %; (**e**) 40 wt % ASF, 6.0 wt %, and (**f**) 50 wt % ASF, 7.5 wt %.

Figure 4 gathers the field emission scanning electron microscopy (FESEM) images for PBS/ASF/MLO composites with increasing ASF content. Figure 4a shows the fractured surface of neat PBS under impact conditions. Obviously, this surface appears to be rough due to deformation during impact. As it has been previously described, a threshold at 30 wt % ASF can be detected. Below 30 wt % ASF, the compatibilizing effect of MLO seems to be clear, thus leading to a progressive increase in mechanical ductile properties. Over 30 wt %, the MLO content is not enough to provide good compatibilization and this suggested poor polymer/particle cohesion. This different behavior can be explained by the following FESEM study. Figure 4b shows the fracture surface of the PBS/ASF/MLO composite with 10 wt % ASF (and the corresponding MLO content, i.e., 1.5 wt %). Both the matrix and the dispersed particles can be clearly identified. A typical spotted surface of an ASF particle is surrounded by the PBS matrix. As it can be seen (with white ellipses), the gap between the particle and the surrounding matrix is almost non-existent thus indicating the positive effect of MLO on establishing polymer/particle interactions through the reaction of succinic anhydride pendant groups with hydroxyl groups in both PBS (end-chains) and cellulose in ASF [66]. Another phenomenon can be observed in this composite. The PBS matrix shows scattered spherical shapes (white arrows) corresponding to MLO [67]. Similar situation can be found for PBS/ASF/MLO composites with 20 wt % (Figure 4c). In this case, a small gap (white ellipse) in the range of several hundred nanometers can be found between the ASF particle and the surrounding matrix, which also shows some more spherical shapes (white arrows) due to MLO with a typical diameter in the nanoscale range. Figure 4d shows the fracture surface of the PBS/ASF/MLO composite with 30 wt % and 4.5 wt % MLO which shows the best balanced mechanical properties (ductile and resistant). ASF are fully embedded inside the PBS matrix and clear evidences of plastic deformation (filaments) can be seen. Some small ASF particles can be seen with a small gap of 100–200 nm (white ellipse). Nevertheless, composites with 40 wt % ASF show a clear difference with regard to the previous composites with 30 wt % ASF or less. If we observe Figure 4e, the gap between the ASF particle and the surrounding matrix (white ellipse) is noticeably higher of 1–2 μm. This phenomenon indicates an excess ASF particles and MLO is not enough to provide a homogeneous interface, thus leading to poor cohesion, which in turn, is responsible for a decrease in ductile properties. This same behavior can be observed for PBS/ASF/MLO composites with 50 wt % ASF (Figure 4f. It is worthy to note that the PBS content in these composites is only 42.5 wt % so that it is evident the poor cohesion among the surface. The PBS matrix also shows the spherical shapes corresponding to MLO. So that, the FESEM study is in total agreement with the previous mechanical properties thus giving evidence of an ASF threshold which determined if MLO compatibilization is effective or not. For 30 wt % ASF or less, MLOs can provide a homogeneous interface and contribute to compatibilize ASF with the PBS matrix. Over 30 wt % ASF, the amount of filler seems to be extremely high and MLO content is not enough to compatibilize ASF with PBS thue leading to poor cohesion is detected.

### 3.2. Thermal Properties of PBS/ASF/MLO Composites

Differential scanning calorimetry was used to obtain the main thermal transitions of PBS/ASF/MLO composites. Figure S2 in Supplementary Materials shows a comparison of the DSC thermograms of PBS/ASF/MLO composites with different ASF and MLO content. Table 3 gathers the main thermal parameters obtained through DSC characterization. As it can be observed, a slight decrease in the melt peak temperature (*T_m_*) from 119.1 °C down to 113–114 °C can be observed thus indicating that ASF favours crystallization as the crystal structure of cellulose in ASF acts as nucleant during crystallization [68]. Obviously, the normalized melt enthalpy (*ΔH_m_*) decreases with the increasing ASF and MLO content. Nevertheless, taking into account the actual PBS mass in each PBS/ASF/MLO composite, calculation of the degree of crystallinity (*%χ_c_*) leads to slightly higher values with increasing ASF content. So that, in addition to the nucleant effect of ASF, it allows developing higher percentage of crystallinity in combination with MLO, which provides increased chain mobility. For this reason, the *%χ_c_* changes from 57.7% for neat PBS up to values of 62–66% with different ASF content. Similar tendency has been found in some biopolyesters such as PLA and other semicrystalline polymers [34,48]. In particular, similar findings were reported by Calabia et al. for PBS composites with varying cotton fiber loading in the 0–40 wt % range. They reported a clear increasing tendency on crystallinity with increasing cotton fiber loading [68].

**Figure 5:**
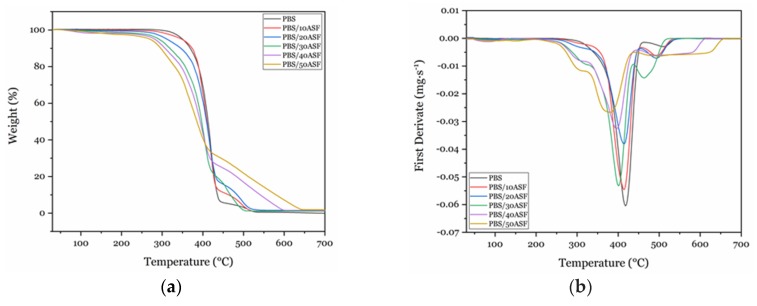
(**a**) thermogravimetric (TGA) degradation curves and (**b**) first derivative (DTG) of PBS/ASF/MLO composites with varying ASF loading.

With regard to thermal degradation at high temperatures, thermogravimetric analysis—TGA gave the main degradation parameters, i.e., *T*_5%_ and *T_max_* which correspond to the temperature for a mass loss of 5% and the temperature for a maximum mass loss rate, respectively. Figure 5a shows the TGA degradation curves of PBS/ASF/MLO composites with increasing ASF content and Figure 5b shows the first derivative that allows identifying the temperature for the maximum mass loss rate. Poly(butylene succinate) degrades in a single step process and its *T*_5%_ is close to 338.1 °C, thus indicating high thermal stability. Almond shell flour, as other lignocellulosic fillers degrades in a complex process with several overlapping stages. The first stage is residual moisture removal at a temperature range of 80–100 °C. Over 250–270 °C, hemicellulose starts its degradation reactions followed by the more thermally stable cellulose domains. Degradation of cellulose and hemicellulose involves complex reactions comprised in the temperature range of 250–370 °C. Regarding lignin, it is worthy to note that its degradation occurs in a wider temperature range from 250 °C up to 450–500 °C [69,70]. In general, as ASF shows lower thermal stability than PBS matrix, the typical TGA curves are moved towards lower temperatures with increasing the ASF content as Figure 5a shows. As shown in Table 4, the *T*_5%_ changes progressively from 338.1 °C for neat PBS down to values of 256.3 °C for the PBS/ASF/MLO composite containing 50 wt % ASF. The same tendency can be found for the temperature corresponding to the maximum mass loss rate (*T_max_*), which is represented in Figure 5b as the peak minimum which is moved towards lower temperature values as it can be quantified in Table 4. In particular, the *T_max_* changes from 414.7 °C for neat PBS to values of 378.7 °C for the PBS/ASF/MLO composite with the highest ASF content (50 wt %). All composites (even that with the highest ASF content of 50 wt %) are thermally stable up to 250 °C which indicates processing can be carried out in a wide temperature window since the melt process of PBS is comprised between 100–120 °C. Poly(butylene succinate) shows high thermal stability as other polyesters but lignocellulosic particles degrade at lower temperatures. Nevertheless, as the melt process of PBS is moderate, it is usually processed at temperatures in the 130–140 °C. As it has been shown by TGA analysis, all composites show thermal stability up to 250 °C. So that, although ASF addition leads to decreased thermal stability, it does not compromise processing and applications of PBS/ASF composites which can find interesting applications in the automotive industry (interior panels), construction and building (fencing, gates, panels, railings, and so on), outdoor furniture parts, etc. [71,72,73].

### 3.3. Thermomechanical Properties of PBS/ASF/MLO Composites.

**Figure 6:**
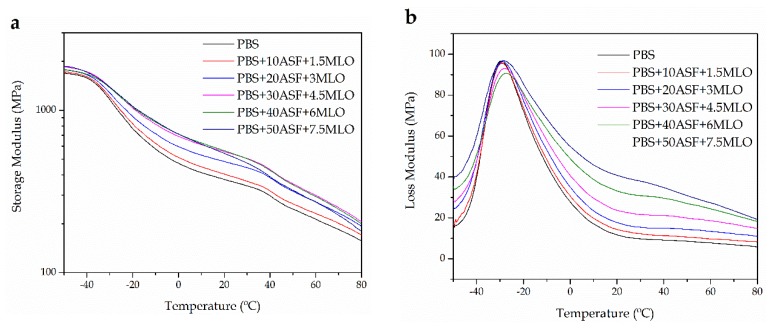

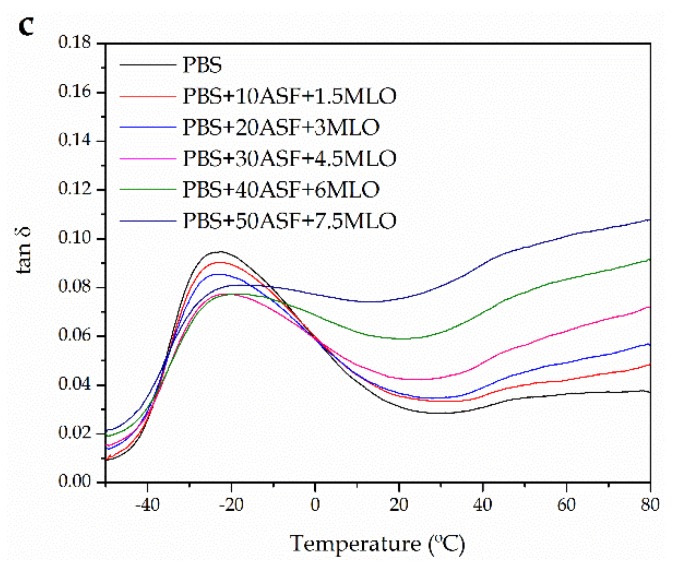
Plot evolution of (**a**) storage modulus (*E’*), (**b**) loss modulus (*E”*), and (**c**) damping factor (*tan* *δ*) of PBS/ASF/MLO composites with varying ASF loading.

Dynamic mechanical thermal characterization—DMTA was used to evaluate the influence of temperature on mechanical behavior of PBS/ASF/MLO composites. Figure 6a shows the plot evolution of the storage modulus (*E’*) as a function of temperature for PBS/ASF/MLO composites with increasing ASF loading. At low temperatures of −50 °C, all composites show similar storage modulus and the difference in behavior can be observed over the glass transition temperature, *T_g_* (located in the −40/−10 °C range). At room temperature the material with the lowest stiffness is neat PBS and, as the ASF increases, the characteristic DMTA curve is moved to higher *E’* values. This behavior is typical in dynamic tests with other polymer/natural filler composites [74]. PBS is a viscoelastic polymer and as the damping factor represents the ratio between the loss modulus (*E”*) to the storage modulus (*E’*), it is possible to see in Figure 6b that over the glass transition process, the loss modulus increases with increasing ASF due to internal friction between PBS polymer chains and ASF particles. For this reason, above the glass transition process, the damping factor also increases with the ASF content. Regarding the damping factor (*tan* *δ*), (Figure 6c), the maximum damping factor corresponds to PBS and decreases as the ASF content increases. The peak maximum corresponding to the damping factor, can be assigned to the glass transition temperature (*T_g_*) of the PBS-rich phase. Neat PBS shows a *T_g_* value of about −23 °C and this is slightly moved to −20 °C by the addition of MLO to PBS/ASF/MLO composites with up to 30 wt % PBS. This indicates somewhat interaction as observed by FESEM. Nevertheless, the change in *T_g_* is not remarkable.

In addition to dynamic mechanical thermal characterization (DMTA), thermomechanical analysis was used to study the thermal stability of PBS/ASF/MLO composites. Table 5 summarizes the values of the coefficient of linear thermal expansion (CLTE) both below and above the *T_g_*. Obviously, the *CLTE* values are lower below the *T_g_* since the material behaves as rigid and temperature does not affect in a great extent to a change in dimension. On the other hand, the *CLTE* values measured above the *T_g_*, are remarkably higher as the material behaves as a softened/plastic material. In particular, neat PBS possesses a *CLTE* of 84.4 μm m^−1^ °C^−1^ and 220.3 μm m^−1^ °C^−1^ below and above the *T_g_*, respectively. As it can be expected, the addition of a lignocellulosic filler leads to improved dimensional stability. Thus, for CLTE values measured below the *T_g_*, all PBS/ASF/MLO composites show lower values compared to neat PBS. Nevertheless, the dimensional stabilization that ASF can provide to PBS/ASF/MLO composites is much evidence by analysing the CLTE values measured above the *T_g_* that changes from 220.3 μm m^−1^ °C^−1^ (neat PBS) down to values of 149.8 μm m^−1^ °C^−1^ for composites with 50 wt % ASF [75].

## 4. Conclusions

In this work, high environmentally-friendly composites with a PBS matrix and a lignocellulosic waste from almond shell, were successfully manufactured by extrusion/compounding followed by injection moulding. Almond shell waste, in the form of powder (ASF), was added in the 0–50 wt % range and, to improve polymer/particle interactions, a vegetable oil-derived compatibilizer, namely maleinized linseed oil, was used with a constant ratio regarding the ASF content. These composites offer a wood like color and can be used as wood plastic composites. Composites containing 30 wt % ASF and 4.5 wt % MLO, offer the best balanced properties. In particular, it shows the maximum impact strength with balanced modulus and elongation at break. The obtained results suggest an ASF threshold of 30 wt %. Below this threshold MLO can provide improved PBS/ASF interactions as confirmed by field emission scanning electron microscopy. Over 30 wt % ASF, composites are more brittle and interface phenomena are less intense thus leading to poor material cohesion, therefore indicating poor compatibilization of MLO due to high ASF content and MLO saturation. As PBS is a very flexible material, the PBS/ASF/MLO composites obtained in this work, represent an interesting technical solution to low-medium mechanical properties composites with potential uses as wood plastic composites.

## Figures and Tables

**Table 1:** Formulations and sample coding of poly(butylene succinate)/almond shell flour/ maleinized linseed oil (PBS/ASF/MLO) composites.

Sample Code	PBS (wt %)	ASF (wt %)	MLO (wt %)
PBS	100	0	0
PBS + 10 ASF + 1.5 MLO	88.5	10	1.5
PBS + 20 ASF + 3 MLO	77	20	3
PBS + 30 ASF + 4.5 MLO	65.5	30	4.5
PBS + 40 ASF + 6 MLO	54	40	6
PBS + 50 ASF + 7.5 MLO	42.5	50	7.5

**Table 2:** Summary of the main mechanical properties of PBS/ASF/MLO composites obtained by tensile, hardness and impact tests.

Sample Code	Maximum Tensile Strength, *σ_t_* (MPa)	Tensile Modulus, *E_t_* (MPa)	Elongation at Break, *ε_b_* (%)	Shore D Hardness	Impact Strength (J m^−2^)
PBS	31.5 ± 0.9	417 ± 21	215.6 ± 16.5	60.1 ± 0.5	16.5 ± 0.8
PBS + 30 ASF [38]	14.8 ± 0.5	790 ± 56	6.3 ± 0.9	71.2 ± 0.3	1.8 ± 0.3
PBS + 10 ASF + 1.5 MLO	24.6 ± 0.2	561 ± 29	17.0 ± 0.6	66.7 ± 0.7	5.4 ± 0.4
PBS + 20 ASF + 3 MLO	18.6 ± 0.4	601 ± 63	20.7 ± 1.3	66.9 ± 0.9	3.9 ± 0.9
PBS + 30 ASF + 4.5 MLO	13.8 ± 0.3	535 ± 51	25.8 ± 1.0	67.2 ± 0.2	3.8 ± 0.5
PBS + 40 ASF + 6 MLO	9.3 ± 0.4	465 ± 76	16.3 ± 0.7	65.3 ± 0.5	2.6 ± 0.2
PBS + 50 ASF + 7.5 MLO	7.1 ± 0.2	364 ± 47	16.4 ± 1.0	63.3 ± 0.5	2.6 ± 0.1

**Table 3:** Main thermal properties of PBS/ASF/MLO composites with varying ASF content, obtained by differential scanning calorimetry (DSC) analysis.

Sample Code	Melt Enthalpy, *ΔH_m_* (J g^−1^)	Melt Peak Temperature, *T_m_* (°C)	*χ_c_* (%)
PBS	65.1 ± 1.7	119.6 ± 0.9	57.7 ± 1.7
PBS + 10 ASF + 1.5 MLO	61.0 ± 2.4	113.8 ± 1.2	62.4 ± 2.5
PBS + 20 ASF + 3 MLO	52.5 ± 1.2	113.8 ± 2.1	61.8 ± 1.4
PBS + 30 ASF + 4.5 MLO	47.8 ± 2.8	113.9 ± 1.9	66.2 ± 3.9
PBS + 40 ASF + 6 MLO	37.2 ± 0.9	113.8 ± 0.9	62.5 ± 1.5
PBS + 50 ASF + 7.5 MLO	31.0 ± 1.6	114.1 ± 1.7	66.1 ± 3.4

**Table 4:** Main thermal degradation parameters of PBS/ASF/MLO composites with varying ASF content, obtained by thermogravimetric analysis (TGA).

Sample Code	*T*_5%_ (°C)	*T_max_* (°C)	Residual Weight (%)
PBS	338.1	414.7	0.39
PBS + 10 ASF + 1.5 MLO	338.0	414.9	1.14
PBS + 20 ASF + 3 MLO	305.3	414.2	1.58
PBS + 30 ASF + 4.5 MLO	295.8	407.6	0.68
PBS + 40 ASF + 6 MLO	272.7	395. 7	1.39
PBS + 50 ASF + 7.5 MLO	256.3	378.7	1.86

**Table 5:** Variation of the coefficient of linear thermal expansion (*CLTE*) below and above the glass transition temperature (*T_g_*) of PBS/ASF/MLO composites with varying ASF loading.

Sample Code	*CLTE* (μm m^−1^ °C^−1^) Obtained by TMA		Thermal Parameters Obtained by DTMA	
	Below *T_g_*	Above *T_g_*	*T_g_* (°C)	*tan* *δ*
PBS	84.4	222.3	−22.9	0.094
PBS + 10 ASF + 1.5 MLO	85.11	193.6	−22.8	0.090
PBS + 20 ASF + 3 MLO	81.8	166.9	−23.0	0.085
PBS + 30 ASF + 4.5 MLO	74.9	167.1	−21.6	0.077
PBS + 40 ASF + 6 MLO	83.5	168.0	−18.2	0.077
PBS + 50 ASF + 7.5 MLO	81.7	149.8	−17.9	0.081

## Supplementary Materials

The following are available online at http://www.mdpi.com/1996-1944/11/11/2179/s1, Figure S1: Comparative plot of the stress (*σ*)–strain (*ε*) curves of PBS/ASF/MLO composites with varying ASF content., Figure S2: Comparative plot of the DSC thermograms of PBS/ASF/MLO composites with varying ASF content.
