# Supplementary material for: The Effect of Varying Almond Shell Flour (ASF) Loading in Composites with Poly(Butylene Succinate (PBS) Matrix Compatibilized with Maleinized Linseed Oil (MLO)
Source: Materials (Basel). 2018 Nov 3;11(11):2179. doi: 10.3390/ma11112179 (PMC6265868; doi:10.3390/ma11112179)
Supplement: Supplementary file 1 [file materials-11-02179-s001.pdf]

materials

Supplementary Information

The effect of varying almond shell flour (ASF) loading in composites with poly(butylene succinate) – (PBS) matrix compatibilized with maleinized linseed oil – MLO

Patricia Liminana [1], Luis Quiles-Carrillo [1]*, Teodomiro Boronat [1], Rafael Balart [1] and Nestor Montanes [1]

[1] Technological Institute of Materials (ITM), Universitat Politècnica de València (UPV), Plaza Ferrándiz y Carbonell 1, 03801 Alcoy, Spain Affiliation 1; patligre@mcm.upv.es (P.L); luiquic1@epsa.upv.es (L.Q.-C.); tboronat@dimm.upv.es (T.B); rbalart@mcm.upv.es (R.B); nesmonmu@upvnet.upv.es (N.M.).

* Correspondenceluiquic1@epsa.upv.es; Tel.: +34-966-528-433

A1. Stress-strain curves of PBS/ASF/MLO composites with varying ASF content.

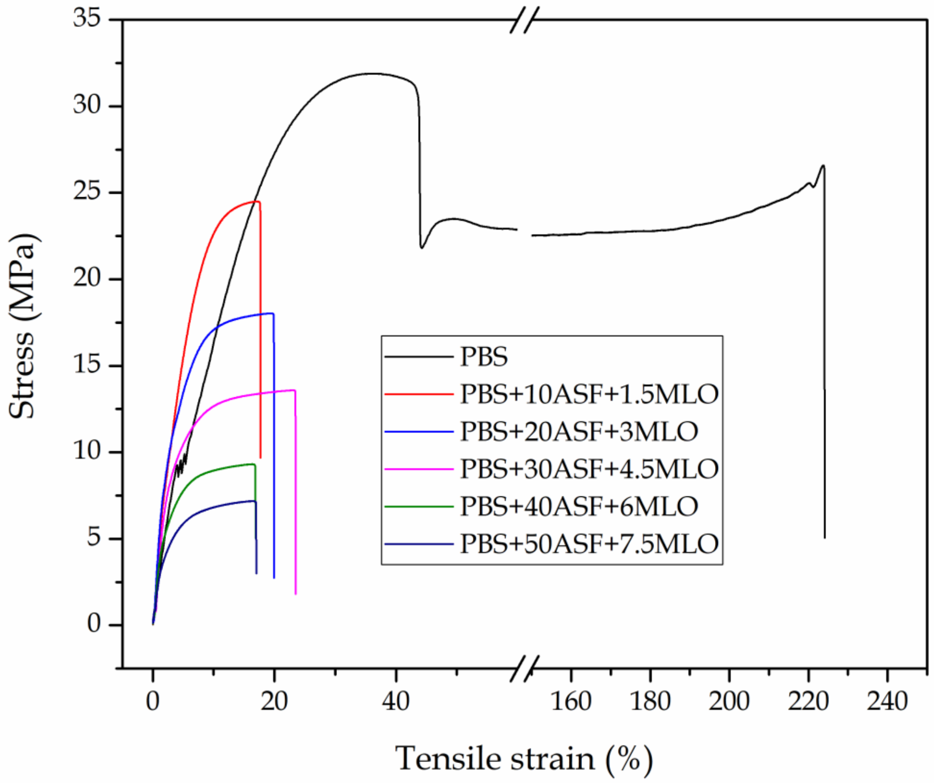

Figure S1. Comparative plot of the stress (σ) –strain (ε) curves of PBS/ASF/MLO composites with varying ASF content.

A2. Differential scanning calorimetry (DSC) thermograms of PBS/ASF/MLO composites with varying ASF content.

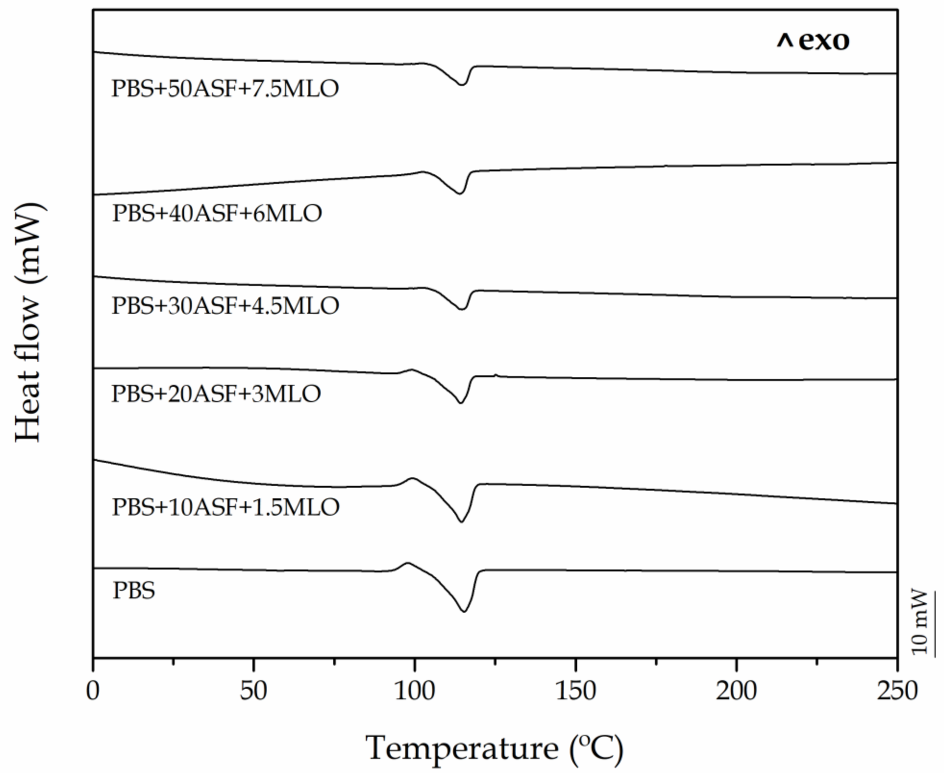

Figure S2. Comparative plot of the DSC thermograms of PBS/ASF/MLO composites with varying ASF content.

© 2018 by the authors. Submitted for possible open access publication under the terms and conditions of the Creative Commons Attribution (CC BY) license (http://creativecommons.org/licenses/by/4.0/).
